# Authors’ Reply: Same Name, Moving Target: Model Version Drift in Syntheses of Large Language Models for Mental Health

**DOI:** 10.2196/112028

**Published:** 2026-10-15

**Authors:** Benjamin Johnson, Janni Leung, Gary Chung Kai Chan

**Affiliations:** 1 The School of Psychology, National Centre for Youth Substance Use Research, 31 Upland Road, Brisbane, Queensland, 4067, Australia, 61 429894154

**Keywords:** large language models, generative artificial intelligence, generative AI, GenAI, mental health, primary health care, screening, clinical decision support, psychotherapy, digital health, eHealth, PRISMA

 We thank Zhang [1] for highlighting model-version drift as an important issue in the synthesis of evidence on large language models (LLMs). We agree that labels such as “GPT-4” may refer to different model versions or snapshots with different behavior and performance.

This issue is not unique to LLM research. In health research, interventions included in systematic reviews and meta-analyses are rarely exactly the same across studies. LLMs add another layer of complexity because models reported under the same name may change over time.

In our review [2], we grouped models as closely as possible based on information reported in the primary studies. Our data extraction captured model version and evaluation date, as reported in Multimedia Appendix 2 of our original article. The appendix includes snapshot-level identifiers for several studies and an evaluation-date field for every included study. Where a primary study did not report an exact model version or evaluation date, we did not infer the missing detail; evaluation dates absent from the primary report were recorded as “not explicitly reported.” Model-version drift may contribute to the substantial between-study heterogeneity observed in our meta-analysis. High *I*² values and wide prediction intervals reflect substantial variability, but cannot be used to determine how much is attributable specifically to model version rather than prompting strategies, populations, tasks, data sources, or outcome definitions. The pooled estimates therefore should not be interpreted as the performance of a single, stable model, but as an average estimate across several closely related models. Our finding that GPT-4 (OpenAI) had higher pooled sensitivity was descriptive: the model groups were pooled separately, no formal between-group test was performed, and the finding should not be interpreted as evidence that GPT-4 was superior to GPT-3.5.

Better reporting is needed. Future studies should report the exact model and version, date of testing, access route, prompts, and relevant model settings. TRIPOD-LLM (Transparent Reporting of a Multivariable Prediction Model for Individual Prognosis or Diagnosis–LLM) provides an important framework for improving reporting and reproducibility [3] and would allow future systematic reviews to better examine model-version effects.

However, improved reporting alone will not address the broader evidence gap. LLMs are already being used in practice while the evidence base remains limited and rapidly changing. A wider range of study designs, including pragmatic trials and well-designed observational studies using causal inference methods, will be needed to estimate the real-world effects of LLM use in mental health care [4]. Evidence synthesis will also need to become more timely. AI-assisted screening and increasingly automated systematic review systems may support more frequent updating as new models and evidence emerge [5].

The commentary therefore highlights a broader challenge: we need both better reporting of what is being evaluated and research and evidence-synthesis methods that can keep pace with rapidly changing technology.

## Abbreviations

LLM: large language model
TRIPOD-LLM: Transparent Reporting of a Multivariable Prediction Model for Individual Prognosis or Diagnosis–Large Language Model
